# Chicago Dental Society

**Published:** 1884-08

**Authors:** A. E. Baldwin


### CHICAGO DENTAL SOCIETY.

### JUNE MEETING.

REPORTED FOR THE INDEPENDENT PRACTITIONER BY A. E. BALDWIN, M. D.

President Harlan in the chair.

The subject of the evening was "Dentition."

A concise and well written paper on the subject was first presented by Dr. P. J. Kester, who commenced by saying, that in the past men were ruled by prejudices and opinions, but in the present we think and investigate, and as theories are proved or disproved, they are accepted or rejected.

By "dentition" we mean that process by which the tooth is impelled to leave its crypt in the alveolus, and is propelled through the jaw and gums, and makes its appearance in the mouth. This name has been used as a cloak to hide more professional ignorance than that concerning any other one physiological process of the human economy.

The people at large, and many of the profession, believe that teething causes nearly all diseases of childhood or infancy. The writer considered the subject under two heads: First, dentition as a physiological process; second, dentition and its relation to disease. Concerning development of the germ he commended Dr. Dean's "Dental Follicle," and in regard to calcification Dr. Tomes' works; but as to the eruptive process, *per se*, information is meager, opinions varying from that expressed by an old edition of Fox & Harris, which says "a passage for the teeth is opened by the process of ulceration," to the latest life force theory, as laid down by Dr. Black in his admirable lectures on the resorption of tissue by the action of the connective tissue cells taking upon themselves the office of odontoclasts.

As to the relation of dentition to disease, the essayist said it is claimed that nearly all diseases of infancy and childhood are caused by this process—the older the authority the greater the number enumerated—but all seem to dwell upon the fact that as dentition is in progress it must be the causative influence of all, or nearly all, infantile diseases.

After enumerating many authorities, the writer said that he is led to believe that improper or insufficient food and dress, poor sanitary conditions, decaying organic matter, bad drainage, etc., etc., are more to blame, in a majority of cases, than is dentition. He quoted Dr. Little, of the Rochester Orphan Asylum, as stating that the principal causes of enteric diseases are over feeding, poor diet, and bad sanitary conditions, and Dr. Jacobi as asserting that these diseases come from over feeding, hot and foul air, but never from teething.

In concluding, the writer, after quoting largely from the many standard authors, summed up with these thoughts: There is no doubt there are many disturbances in the mouths of children during dentition, but we believe them to be either symptomatic of some disease of the digestive or nervous system, or due to accidental or local causes. If it be true that dentition, being a physiological process, has no direct influence on the general system, with the exceptions noted, it is our duty as professional men to teach our patients the danger of attributing diseases to it: for in our opinion, the quota of presidential possibilities is lessened every year by the belief that dentition is the cause of disease.

DISCUSSION.

*Dr. Marshall*—Said that he regarded the process of dentition as somewhat analogous to fœtal expulsion in childbirth, for in both the process is hastened by the contraction of the adjacent tissues. He stated that while he regarded dentition as physiological, he felt convinced that he had seen many cases where the diseased condition of the child rapidly subsided under the judicious use of the lancet on the gums.

*Dr. Noyes*—Thinks we must consider the process as a physiological one, and that when we find those disturbances commonly ascribed to dentition, it is, in his experience, easy to see that they are at least greatly aggravated by feebleness and poorly-nourished systems. In such cases prompt relief is usually given by attention to the gum.

*Dr. Marshall*—Thought that, as the cerebro-spinal system is very early in its development, we may find in the existence of small, persistent irritation centers, the cause of the common ills of dentition, and the reason for the excessive death rate in children.

*Dr. Reid*—Has given the subject much thought and attention, and is led to believe that the judicious use of the gum lancet will relieve many, if not all cases of irritation.

*Dr. Newkirk*—Thought that in every process of development there is a demand for a certain expenditure of nervous or vital force. Thus, in a rapidly-growing child this may be excessive; so in a dentition that is developing too rapidly, pain and disturbances of various kinds may be the direct result.

*Dr. Brophy*—Commended the paper very highly, but said that some of the statements were not quite definite enough. In many cases, commonly credited to dentition, he had found the condition to be caused by intestinal irritation from the ingestion of starchy foods, which, before dentition, cannot be digested, owing to absence of ptyalin in the saliva. His observation leads him to think that the irritation of the developing tooth is not only at the cutting edge, but also at the formative end. The tooth lies in direct contact with the nerve, and in many cases the irritation is bad enough to produce ills resulting in the death of the child. He has found that cutting the gums gives almost instant relief, by letting out the congested blood. He desired to correct the impression that the cicatrix is more dense and harder than natural tissue. He also thinks that children often suffer, and that severely, from neuralgia, and that many diseases are caused directly from irritation in dentition.

*Dr. Gregory*—Thinks that in a child properly cared for as to clothing and diet, dentition is accomplished perfectly normally.

*Dr. Crouse*—Expressed his pleasure at the independent spirit manifested in the paper, and said that with many of the ideas he was in hearty accord. He thought that many times dentition was made to carry burdens properly belonging elsewhere. Thinks that diet has more to do with the ills of childhood than we think. Many things are fed to children that would cause irritation to the healthy stomach of an adult. He thinks that should children be warmly dressed, and fed properly and at stated intervals, as a rule they would be markedly benefited. Thought that in many cases the judicious use of the lancet was very beneficial. He could not endorse the idea of severe neuralgia in children.

*Dr. Mariner*, of Ottawa—Cited a case where a physician in his city lanced the gums of a child, and the result was a death from hemorrhage. Since that time gum lancing has not been as popular with them.

After some other desultory discussion, the Society adjourned to meet in July.

